# Effect of β-Cyclodextrin on the Aggregation Behavior of Sodium Deoxycholate and Sodium Cholate in Aqueous Solution

**DOI:** 10.3390/molecules30102197

**Published:** 2025-05-17

**Authors:** Vesna Tepavčević, Zita Farkaš Agatić, Ana Pilipović, Gorana Puača, Mihalj Poša

**Affiliations:** Department of Pharmacy, Faculty of Medicine, University of Novi Sad, Hajduk Veljkova 3, 21000 Novi Sad, Serbia; zita.farkas@mf.uns.ac.rs (Z.F.A.); ana.pilipovic@mf.uns.ac.rs (A.P.); gorana.puaca@mf.uns.ac.rs (G.P.); mihaljp@uns.ac.rs (M.P.)

**Keywords:** β-cyclodextrin, sodium deoxycholate, sodium cholate, inclusion complex, micellization

## Abstract

This study investigated the influence of β-cyclodextrin (βCD) on the micellization behavior of two bile salt surfactants, sodium deoxycholate (NaDC) and sodium cholate (NaC), in aqueous solutions. Tensiometry, conductometric, and spectrofluorimetric techniques were employed to determine critical micelle concentrations (CMCs) in the presence of varying concentrations of βCD, as well as in the presence of inorganic salts (NaCl and CsCl). The results showed that βCD forms inclusion complexes with both bile salts, leading to an increase in their CMCs, consistent with a competitive interaction between micelle formation and complexation. The inclusion constants, determined graphically, revealed stronger complexation for NaDC than NaC, attributed to differences in hydrophobic surface area. Salt addition decreased the CMC of both surfactants, with CsCl having a more pronounced effect. However, salt presence also modulated the inclusion complex formation, suggesting specific ion effects influence the availability and behavior of βCD. These findings contribute to the understanding of bile salt–cyclodextrin interactions and their modulation by electrolytes, with implications for drug delivery and supramolecular chemistry.

## 1. Introduction

**Figure 1:**
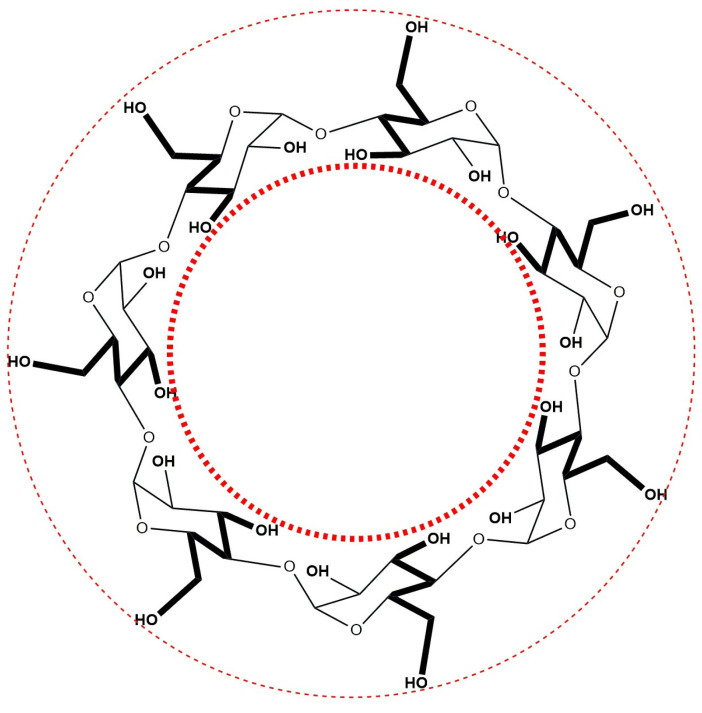
Structure of β-cyclodextrin (βCD).

Cyclodextrins (CDs) are cyclic oligosaccharides composed of glucopyranose units linked by 1,4-glycosidic bonds. The three primary types of cyclodextrins—α, β, and γ—consist of six, seven, and eight glucopyranose units, respectively [1]. Structurally, cyclodextrins exhibit a truncated cone shape characterized by a hydrophobic internal cavity and a hydrophilic external surface, arising from the arrangement of their pyranose units. The hydrophobicity of the cavity is attributed to the presence of skeletal carbon atoms and ethereal oxygen, which facilitates the encapsulation of hydrophobic molecules of appropriate size within the cavity [2]. The hydrophobic cavity creates a space that allows poorly water-soluble molecules to protect their most hydrophobic regions. Contact between such a poorly soluble compound and a cyclodextrin in an aqueous environment can result in complexation. The hydrophilic exterior of cyclodextrins enables these complexes to be water-soluble. Therefore, CDs are known for forming inclusion complexes with various molecular species through several driving forces, with hydrophobic interactions playing a significant role. Of the three most important cyclodextrins, βCD (with a cavity diameter of 6.0–6.4 Å) is the cyclodextrin of most interest because its cavity size allows for the best spatial fit for many common guest moieties [3] (Figure 1).

Cyclodextrins (CDs) are versatile excipients that have been widely studied for their diverse applications in the pharmaceutical field. In pharmaceutical formulation, cyclodextrins are used to enhance the solubility of poorly water-soluble drugs, provide controlled drug release, mask unpleasant tastes or odors, stabilize unstable compounds, and modify pharmacokinetic properties such as absorption rates. This versatility makes cyclodextrins valuable in designing more efficient and patient-friendly pharmaceutical formulations [4]. Drugs such as aripiprazole, mitomycin, diclofenac sodium, chlordiazepoxide, meloxicam, alfaxalone, cisapride, indomethacin, insulin (nasal spray), and omeprazole have been reformulated using cyclodextrins for both commercial and health benefits [5,6,7].

Surfactants are among the most attractive guest molecules for CDs due to their structural complementarity with the cavity of CDs, their broad range of applications, and their commercial availability. They represent another important group of excipients used in pharmacy, primarily due to their ability to spontaneously form micelles in solution and facilitate the solubilization of hydrophobic compounds in aqueous environments. These abilities result from the amphiphilic structure surfactants. The classical surfactants are typically comprised of two distinct regions: a hydrophobic tail and a hydrophilic head. The hydrophobic tail, often composed of long hydrocarbon chains, repels water and has affinity for nonpolar substances. In contrast, the hydrophilic head, which may be ionic or nonionic, interacts favorably with water, facilitating solubility and dispersion [8,9,10,11,12,13,14,15].

**Figure 2:**
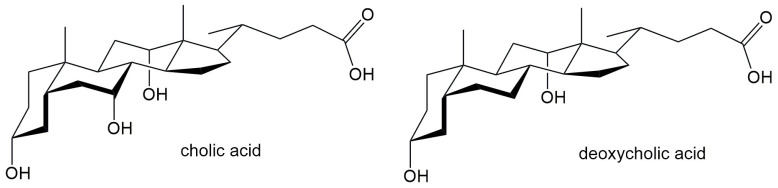
Bile acids whose Na salts are applied in interaction with CD.

Salts of bile acids, however, represent unconventional surfactants, consisting of a steroidal backbone with a hydrophobic side chain and variable hydroxyl groups that impart hydrophilic properties. The spatial geometry of bile salts, with hydrophilic groups on one side of the steroid skeleton and hydrophobic groups on another side of the steroid skeleton (Figure 2) causes their amphiphilic nature. Derived from bile acids and naturally occurring in vertebrates, they act as biosurfactants. Among the bile salts, the most often studied are sodium cholate (NaC) and sodium deoxycholate (NaDC). Both compounds are widely utilized in biological and pharmaceutical research due to their ability to form micelles and interact with lipids and hydrophobic molecules [16,17,18,19,20].

The critical micellar concentration (CMC) of surfactants is the concentration of surfactants in solution at which they create self-assembled molecular clusters called micelles [21,22]. Since the interactions involved in surfactant self-assembly (hydrophobic, electrostatic, and/or steric interactions) are relatively weak compared to chemical bonds, modulation of self-assembly can be readily achieved by varying solvent conditions, e.g., by changing pH, ionic strength, or temperature, or by adding cosolutes or cosolvents. Cyclodextrins (CDs) are among the solutes that are known to affect the association of surfactants in aqueous solutions [23]. The complexation of surfactants by cyclodextrins produces a change in their physicochemical properties, because of the insertion of the hydrophobic part into the cyclodextrin cavity. Depending upon the size of the CD cavity and the chemical nature of the surfactant, a variety of complexes may form, the stoichiometry of which is 1:1 (one CD molecule per one surfactant molecule), 2:1 (two CD molecules per one surfactant molecule), etc. [24].

Previous studies have reported that β-cyclodextrin can form inclusion complexes with bile salts such as sodium cholate and sodium deoxycholate, affecting their micellization behavior [23,24]. These interactions are influenced by both the hydrophobic characteristics of the guest molecule and the size and polarity of the cyclodextrin cavity [25]. In particular, trihydroxy bile salts tend to form weaker complexes compared to their dihydroxy counterparts, likely due to differences in spatial configuration and hydration [26]. Thermodynamic studies also suggest that the inclusion process is governed by both enthalpic and entropic contributions, depending on the nature of the guest molecule [27]. Although several studies have explored the formation of inclusion complexes or the micellization behavior of bile salts individually, few have systematically investigated the interrelationship between these two processes, especially under varying ionic conditions. Our work aimed to fill this gap by quantifying both micellization and complexation in the same experimental system.

The objective of this study was to investigate the effect of hydrophobic surface area and molecular rigidity of bile acid anions (Figure 2), in comparison to conventional surfactants, on the equilibrium constants related to the formation of inclusion complexes with β-cyclodextrin (βCD) at 298.15 K. In addition, the influence of alkali metal ions (Na^+^ and Cs^+^), known to affect hydrophobic interactions [28], was examined with respect to both inclusion complex formation and micellization in aqueous media. These phenomena were studied using tensiometry, conductometry, and spectrofluorimetric techniques, allowing for a comparative analysis of the factors that govern self-assembly and host–guest interactions in the systems under investigation.

## 2. Results

### 2.1. Interaction of β-Cyclodextrin and Bile Salts in Aqueous Solution Without Additives

The tensiometric method is primarily employed as a preliminary technique to assess whether the surface tension is altered during the saturation of the aqueous solution–air interface with surfactants in the presence of the investigated cyclodextrin. If the saturation surface tension changes in the presence of cyclodextrin, it means that the formation of micelles and the formation of the inclusion complex between surfactants and cyclodextrin cannot be treated as independent thermodynamic processes, i.e., as elementary thermodynamic processes. Saturation surface tension changes in the presence of cyclodextrin also imply that the inclusion complex, alongside the free surfactant, participates in the adsorption at the aqueous solution–air interface. For nonionic surfactants with a critical micellar concentration (CMC) value lower than 1 mM (as well as ionic surfactants with a long alkyl chain, e.g., C16), if the inclusion complex is not incorporated into the boundary surface, there is an initial plateau on the surface tension dependence curve on the total concentration of surfactants. The lack of such a plateau indicates that the inclusion complex also has surface properties. No plateau is observed for surfactants with relatively high CMC values.

**Figure 3:**
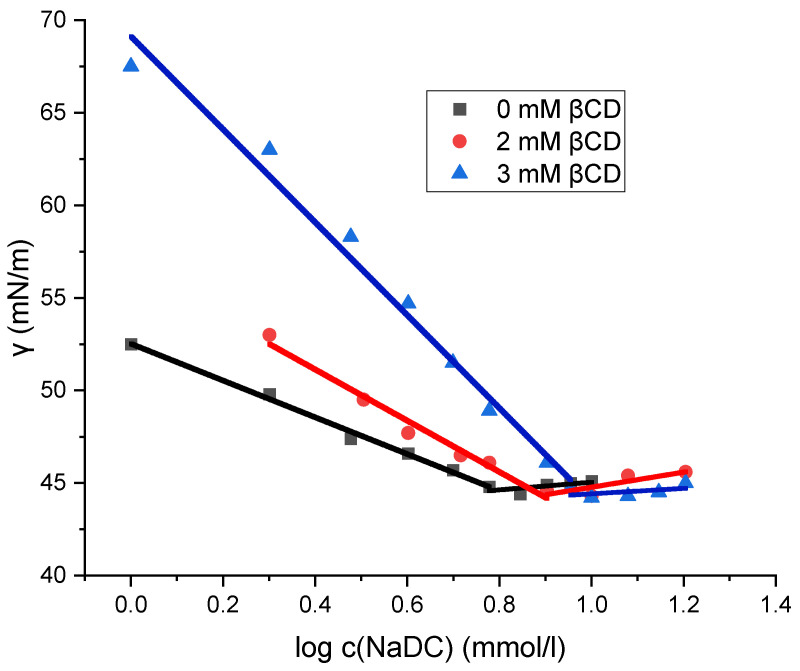
Plot of surface tension as a function of the logarithm of sodium deoxycholate concentration. The plot shown is a representative example from one of five independent measurements.

**Figure 4:**
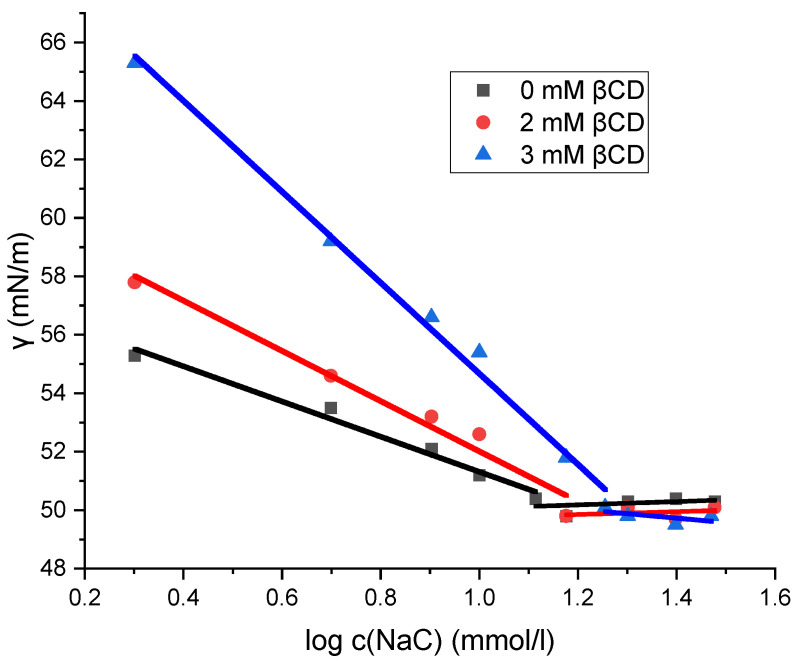
Plot of surface tension as a function of the logarithm of sodium cholate concentration. The plot shown is a representative example from one of five independent measurements.

The micellization of sodium deoxycholate (NaDC) and sodium cholate (NaC) in the absence and presence of β-cyclodextrin (βCD) at 298.15 K was investigated by the tensiometric method. On the isotherms of the surface tension (γ) versus the surfactant concentration for NaDC and NaC, the concentration of the breakpoint in the surface tension isotherm is the CMC, at which there is no further decrease in γ with the further addition of surfactant (Figure 3 and Figure 4). Table 1 shows the critical micellar concentrations (CMCs) of NaDC and NaC in solutions with 0 mM βCD, 2 mM βCD, and 3 mM βCD at 298.15 K, as determined graphically. Miyake determined the critical micelle concentration (CMC) of sodium deoxycholate (NaDC) to be 5 mM in pure water at 293.15 K using tensiometric measurements [29], while Roda reported a CMC value of 13 mM for sodium cholate (NaC) in pure water at 298.15 K [20]. These values are consistent with the CMC data presented in Table 1.

Figure 3 and Figure 4 show that the surface tension values remain unchanged in the solution of sodium cholate (NaC) and in the solution of sodium deoxycholate (NaDC), regardless of whether β-cyclodextrin (βCD) is present or absent. After the breakpoint of curves (Figure 3 and Figure 4), the values of surface tension differ only within the limits of relative uncertainty. Prior to the breakpoint, however, the curves are shifted toward higher surfactant concentrations in the presence of βCD. Although the same amount of the surfactant is incorporated into the interfacial layer at the saturation, a portion of the surfactant in βCD-containing solutions is bound in the inclusion complexes. As a result, increasing the concentration of βCD in solution causes a further shift of the surface tension vs. surfactant concentration curve—before the breakpoint—toward higher surfactant concentrations, i.e., the increase in the concentration of βCD results in higher surface tension values for solutions with the same total surfactant concentration (in the areas before the critical micellar concentration), because an increasing fraction of the surfactant is then in the form of an inclusion complex and a smaller fraction is incorporated into the boundary surface. Therefore, the inclusion complex of surfactant (NaDC or NaC) and βCD is not adsorbed on the aqueous solution–air interface, so the process of self-association and the process of formation of the inclusion complex can be viewed as independent elementary thermodynamic processes [30].

In an aqueous solution of surfactant S and β-cyclodextrin βCD, let there be parallel (competitive) reactions of micelle formation (M) with aggregation number *ν* and formation of the inclusion complex [βCD − S] of 1:1 stoichiometry at constant pressure and temperature:

*v*S ↔ M

S + βCD ↔ [βCD − S]

These equations are obtained for a system of parallel reactions, ensuring the persistence of the critical micellar concentration (CMC) of surfactant S (CMC = *c*_S_ = *const*.). At the critical micellar concentration of surfactant in solution with βCD (CMC_βCD_), the molar concentration of the monomeric surfactant remains unchanged compared to a solution without βCD, at the CMC. The difference CMC_βCD_ − CMC = ΔCMC indicates the amount of surfactant S per unit volume in the inclusion complex with βCD. Notably, the stoichiometry of the inclusion complex plays a pivotal role in determining the inclusion complex equilibrium molar concentration $c_{\left[\beta CD-S\right]}=∆CMC$—i.e., $c_{\left[\beta CD-S\right]}$ represents the equilibrium molar concentration of the inclusion complex (with 1:1 stoichiometry), which is equal to ΔCMC [27]. The equilibrium constant of inclusion complex formation between βCD in the total concentration $c_{\beta CD}^{tot}$ and surfactant S in equilibrium concentration of $CMC=c_{s}$ (i.e., indicating the molar concentration of surfactant S that is not in the form of an inclusion complex and is equal to CMC) can be expressed as follows:

(1) $$K_{\left[\beta CD-S\right]}=\frac{c_{\left[\beta CD-S\right]}}{c_{S}\cdot c_{CD}}=\frac{\Delta CMC}{CMC\cdot (c_{\beta CD}^{tot}-\Delta CMC)}$$

where $c_{CD}$ is the equilibrium molar concentration of βCD, defined as the difference between the total concentration of βCD ($c_{\beta CD}^{tot}$) and the concentration of the inclusion complex ($c_{CD}=c_{\beta CD}^{tot}-∆CMC$).

**Figure 5:**
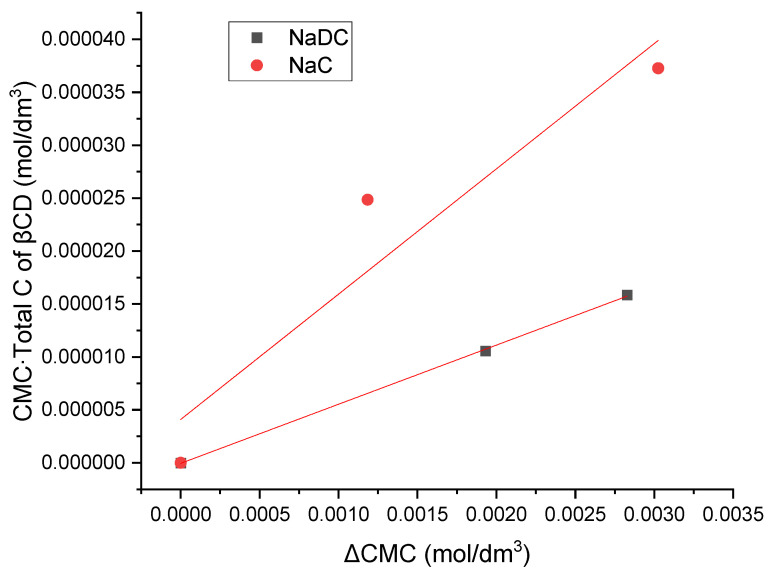
Preliminary graphical method of obtaining the equilibrium constant for the formation of the inclusion complex between bile salts and β-cyclodextrin (βCD). The plot shown is a representative example from one of five independent experiments.

The expression for the equilibrium constant of the inclusion complex formation corresponds to a linear relation of the form *y* = tg*α* ∙ *x*, where the following notations apply: *y* = CMC∙$c_{\beta CD}^{tot}$, *x* = ΔCMC = CMC_CD_ − CMC, and tg*α* = [1/*K*] + CMC. In this formulation, *y* = CMC∙$c_{\beta CD}^{tot}$ increases linearly with an increase in the total concentration of βCD ($c_{\beta CD}^{tot}$) in the aqueous solution of the monocomponent surfactant. Moreover, the slope tan(α) remains constant, since both the CMC of the surfactant and the equilibrium constant *K* for inclusion complex formation are invariant at a given temperature (Figure 5).

The values of the equilibrium constants for the formation of the inclusion complex between bile salts and β-cyclodextrin (βCD), obtained using graphical method, were 3039.51 dm^3^/mol for the complex of βCD with sodium deoxycholate (NaDC) and 927.21 dm^3^/mol for the complex of βCD with sodium cholate (NaC). In previous reports, the equilibrium constants of βCD with trihydroxy bile salts (sodium cholate, taurocholate, and glycocholate) were lower than the equilibrium constants of βCD with dihydroxy bile salts (sodium deoxycholate, sodium taurodeoxycholate and sodium glycodeoxycholate) [31,32].

Besides the interactions between the micelle-building units, the binding of the counterion to the micelle also plays important roles in micelle formation. Such information can be accessed via the surface charge density, which can be measured by specific conductivity (κ) as a function of the concentration of the surfactant (c). The goal of the conductometry measurement is to determine the fraction of counterion binding to the micelle [33].

**Figure 6:**
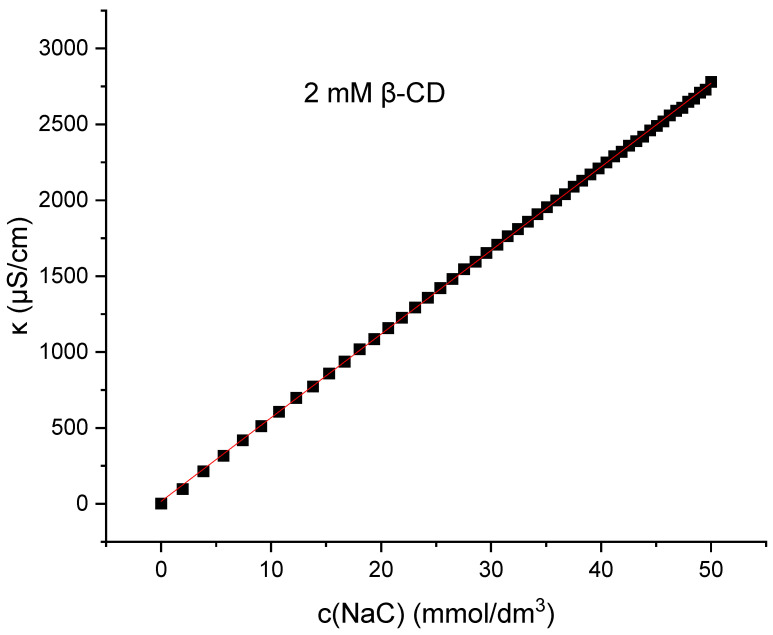
Change in specific conductivity as function of sodium cholate concentration in 2 mM β-cyclodextrin (βCD) solution. The plot shown is a representative example from one of five independent measurements.

As seen in Figure 6, there is no such breakpoint of κ(c) for sodium deoxycholate or sodium cholate in aqueous solutions or in 2 mM β-cyclodextrin aqueous solutions (Supplementary Materials).

### 2.2. Interaction of β-Cyclodextrin and Bile Salts in Aqueous Solution with Sodium Chloride and Aqueous Solution with Cesium Chloride

In aqueous solutions, the addition of salts such as sodium chloride (NaCl) and cesium chloride (CsCl) can alter the micellization behavior of ionic surfactant. The effect of NaCl and CsCl on the micellization behavior of sodium deoxycholate (NaDC) and sodium cholate (NaC) in solutions with and without β-cyclodextrin (βCD) was investigated in this work using spectrofluorometric analysis. Fluorescence spectroscopy was employed specifically in salt-containing systems to probe the microenvironmental polarity within micelles, which is more directly affected by ion-mediated structural reorganization.

**Figure 7:**
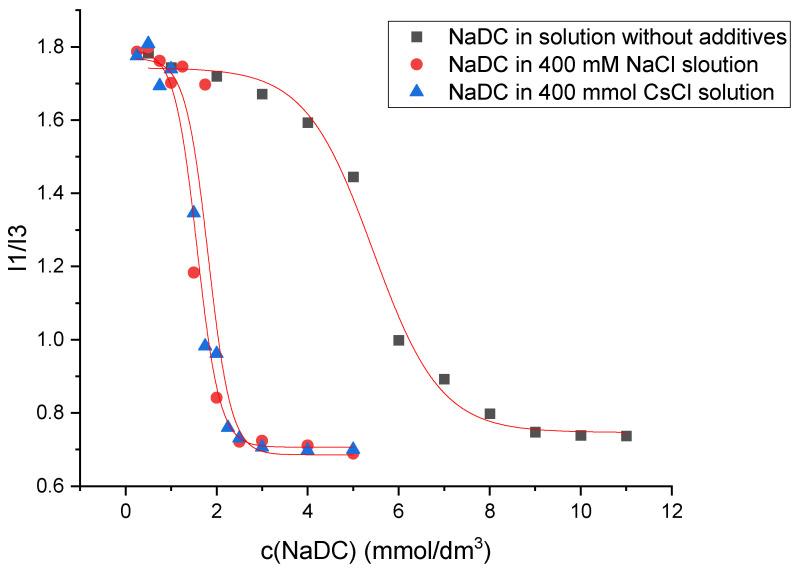
Plot of I_1_/I_3_ ratio against sodium deoxycholate (NaDC) concentration in aqueous solution without additives, in 400 mM NaCl solution, and in 400 mM CsCl solution at a temperature of 298.15 K. The plot shown is a representative example from one of five independent measurements.

**Figure 8:**
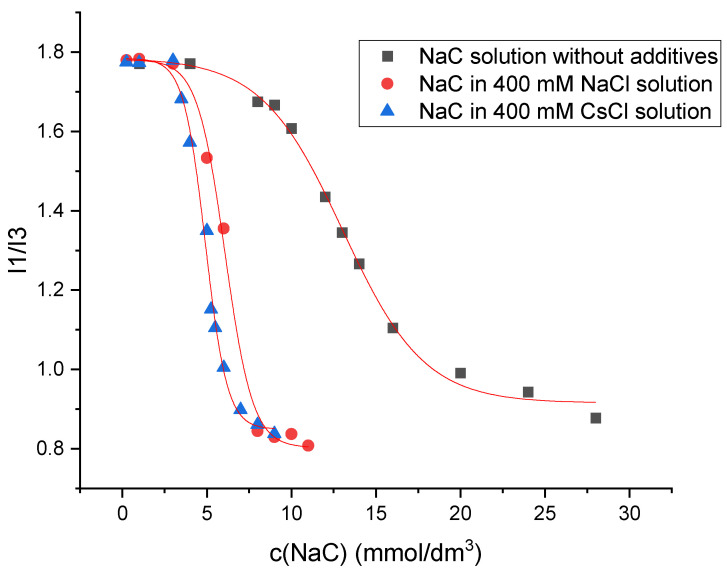
Plot of I_1_/I_3_ ratio against sodium cholate (NaC) concentration in aqueous solution without additives, in 400 mM NaCl solution, and in 400 mM CsCl solution at a temperature of 298.15 K. The plot shown is a representative example from one of five independent measurements.

The determination of the critical micellar concentration (CMC) by spectrofluorometry is an invasive technique that employs pyrene as a hydrophobic probe molecule, which becomes incorporated into the micellar core upon micelle formation. A sharp change in the intensity ratio of the first and third vibronic emission bands of pyrene in the fluorescence measurements indicates the onset of the micellization, causing the decline in the polarity of the pyrene microenvironment [34]. Therefore, this change in the I_1_/I_3_ ratio as a function of surfactant concentration (c) can be used to determine the critical micelle concentration. Figure 7 shows the plot obtained for sodium deoxycholate (NaDC) in aqueous solution without additives, in 400 mM NaCl solution, and in 400 mM CsCl solution at a temperature of 298.15 K, whereas Figure 8 shows the plot obtained for sodium cholate (NaC) in aqueous solution without additives, in 400 mM NaCl solution, and in 400 mM CsCl solution at a temperature of 298.15 K.

Table 2 shows that the CMC values of sodium deoxycholate (NaDC) in solutions with different concentrations of β-cyclodextrin (βCD) increase as the concentration of βCD increases. At all examined temperatures the CMC values increase with the increase in βCD concentration, due to formation of inclusion complexes between βCD and NaDC.

The values of equilibrium constants determined by the graphical method for inclusion complex formation of bile salts and βCD in aqueous solution and aqueous solutions with 400 mM NaCl and 400 mM CsCl are presented in Table 3.

## 3. Discussion

This study investigated the effects of β-cyclodextrin (βCD) on the micellization behavior of two bile salts—sodium cholate and sodium deoxycholate—using tensiometry, conductometry, and fluorescence spectroscopy. By examining the critical micelle concentrations (CMCs) and the formation of inclusion complexes under different ionic conditions, it aimed to clarify how host–guest interactions and specific ion effects modulate aggregation behavior in aqueous media. In addition to the techniques employed in this study, other methods for determining CMCs are reported in the literature and merit consideration. Conductimetry is a non-invasive technique that enables rapid and reliable detection of micellization by monitoring changes in solution conductivity. This method avoids the use of exogenous probes and allows full recovery of the sample, making it suitable for systems where minimal perturbation is required [35]. Another relevant method is UV spectroscopy, which detects micellization-induced spectral shifts of bile salts or other amphiphilic molecules without the addition of fluorescent probes. This approach is particularly useful when chromophoric moieties are inherently present or when indirect estimation is acceptable [36].

While both conductimetry and UV spectroscopy offer significant advantages in terms of non-invasiveness and simplicity, the techniques applied in this work were selected for their specific sensitivity to either interfacial or microenvironmental phenomena. In particular, tensiometry was used for βCD-containing systems because it is the only technique among those applied that can directly reveal whether the βCD–surfactant complex adsorbs at the air–water interface alongside the surfactant itself. This allows for experimental insight into potential synergistic or antagonistic interactions between the complex and the surfactant in the monolayer, which is crucial for correctly interpreting CMC shifts. If the βCD–surfactant complex participates in interfacial adsorption, then the observed change in CMC (ΔCMC) is not equivalent to the equilibrium concentration of the inclusion complex in bulk solution. In contrast, fluorescence spectroscopy was specifically used to study salt-containing systems, as it is highly sensitive to changes in micellar microenvironment polarity—particularly useful when assessing structural reorganization induced by Na^+^ and Cs^+^ ions. This methodological distinction reflects the strengths of each technique in addressing the distinct aspects of the systems under investigation.

The experimental data (Table 1) show that the critical micellar concentration (CMC) of sodium deoxycholate (NaDC) and sodium cholate (NaC) increases in the presence of β-cyclodextrin (βCD) due to formation of inclusion complexes between bile salts and βCD. In an aqueous solution at a CMC, the equilibrium distribution of surfactant between its monomeric, micellar (the bulk solution), and Gibbs interface (aqueous solution–air interface) forms is a constant state, characterized by CMC at constant pressure and temperature. The increase in CMC in the presence of β-cyclodextrin represents the excess amount of surfactant that binds to the inclusion complex, maintaining a constant distribution of monomers among the described states [30].

Although the CMC of sodium deoxycholate (NaDC) and sodium cholate (NaC) increases in the presence of βCD (due to the formation of inclusion complexes), the surface tension values remain unaffected within the limits of determination error (Figure 3 and Figure 4). These findings suggest that the inclusion complexes of βCD formed with the surfactants NaDC and NaC do not incorporate into the interface of bulk solution–air and that only surfactants themselves are accumulating and saturating at CMC the interface. If surfactants in inclusion complexes with βCD do not incorporate into the solution interface, then the principle of detailed balance applies [37], indicating that each equilibrium process occurring (i.e., micelle formation and inclusion complex formation) is thermodynamically independent. The increase in CMC in the presence of βCD (CMC_CD_) reflects the excess amount of surfactant that binds to the inclusion complex, maintaining a constant distribution of monomers among the described states (monomers form bulk, from interface and from micellar state that began formation at critical micelle concentration): ΔCMC = CMC_CD_ − CMC. Thus, even in the presence of a parallel reaction of the inclusion complex formation, the same equilibrium concentration of monomeric surfactant exists and is equal to the CMC, which enables the application of the Equation (1).

**Figure 9:**
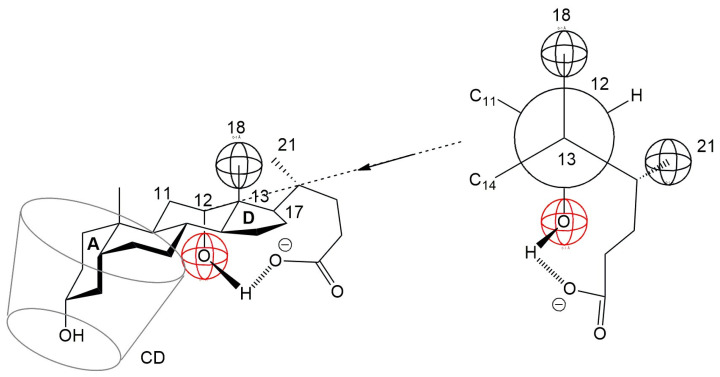
Incorporation of the A ring of the steroid skeleton of the deoxycholic acid anion into the hydrophobic cavity of CD. the D ring is hindered from entering due to the formation of a hydrogen bond between the C17 side chain and the C12 hydroxyl group.

Anions of non-conjugated bile acids, such as cholic acid and deoxycholic acid, are known to form an intramolecular hydrogen bond between the C24 carboxylate group and the C12 α-axial hydroxyl group in both aqueous solution and crystalline states [38]. This intramolecular interaction may increase the steric volume of the D ring, potentially hindering its inclusion into the hydrophobic cavity of β-cyclodextrin (βCD). Consequently, it is plausible that the A ring of the steroid nucleus preferentially enters the βCD cavity, leading to the formation of a 1:1 inclusion complex (Figure 9). This proposed orientation aligns with observations from NMR studies on similar steroidal compounds, where the A ring was found to enter the βCD cavity from the narrow rim, while the D ring remained near the wider rim [39].

Beside the interactions between the micelle-building units, the binding of the counterion to the micelle also plays important roles in micelle formation. Such information can be accessed via the surface charge density, which can be measured by specific conductivity (κ) as a function of the concentration of the surfactant (c). The goal of the conductometry measurement is to determine the fraction of counterion binding to the micelle [21,33]. Transition in specific conductivity is due to the fact that sodium counterions at the micelle surface partially neutralize the net electrical charge of the micelle, and therefore its mobility decreases compared to the state before micellization, when all monomers were ionized. The lack of a breakpoint of κ(c) for sodium deoxycholate and sodium cholate in aqueous solution and in 2 mM βCD aqueous solutions (Figure 6) implies that after the formation of the micelle, the total negative charge is free and the negative charges of the micelle are not neutralized by the oppositely charged ion. Bile acid salts in the vicinity of critical micellar concentration build relatively small micelles—aggregation of several surfactants—compared to classic surfactants, whose aggregation number often exceeds 100 [17,18,40,41,42,43,44,45]. In small micelles of bile acid salts, according to the Kawamura–Small model [46], the carboxylate groups are *trans*-oriented in the discoid micelle (on opposite surfaces of the disk), which does not allow the formation of a Stern bilayer (where the collective property of carboxylate groups and anions is manifested) [47], i.e., binding of counterions, but each carboxylate group behaves individually, as if the studied surfactant is still in the monomeric state and not in the micellar state (of course, there are bile acid anion derivatives where the slope of the κ(c) curve changes with increasing surfactant concentration, for example, some keto derivatives). In the aqueous solution without βCD, with the bile acid anions tested, Cs^+^ ions reduce the CMC somewhat more efficiently than Na^+^ ions (Table 2). In this case, there is no electrostatic shielding of repulsive interactions between anionic groups of surfactants [48], since counterions do not bind to the outer shell of the micelle (Figure 6). Cs^+^ ions as large polarizable cations bind to the hydrophobic surface of the steroid skeleton of bile acid anions [28], thus increasing the total hydrophobic surface of the steroid skeleton, which is of key importance in the entropic mode of self-association (room temperature) [17,42,44,49,50,51,52,53,54,55,56]. On the contrary, Na^+^ ions promote the dehydration of the hydrophobic surfaces of the steroid skeleton, which synergistically affects the association according to the Kawamura–Small model, where the micellar building units are in contact with each other via the β sides of the steroid skeleton (convex surfaces) [12,17]. The selection of Na^+^ and Cs^+^ ions as counterions was intentional to illustrate the effect of ionic size and hydration strength on the aggregation and complexation behavior of bile salts. Sodium ions represent a physiologically abundant and moderately hydrated alkali metal cation, whereas cesium ions are among the least hydrated, with large ionic radii, allowing investigation of specific ion effects on micellization. Potassium ions, although biologically relevant, exhibit intermediate properties and were omitted in this study to preserve clarity and experimental focus. Future work may explore the effect of potassium ions to further elucidate the role of ion-specific interactions in these systems.

**Figure 10:**
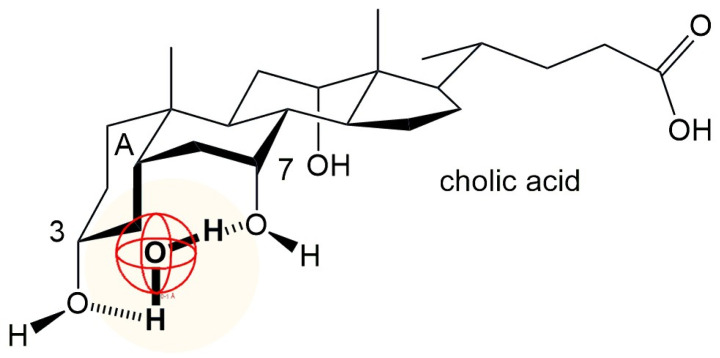
Cooperative binding of water molecules by the C7 and C3 α-oriented OH groups of cholic acid: the degree of hydration of the A ring of the steroid skeleton increases compared to deoxycholic acid (which does not have a C7 OH group).

The equilibrium constant of inclusion complex formation between βCD and sodium deoxycholate (NaDC) is about three to four times higher than the equilibrium constant of inclusion complex formation between sodium cholate (NaC) anion and βCD (Table 3). The steroid skeleton of the NaDC anion, in addition to the convex hydrophobic surface (β side of the steroid skeleton), also has a C7 lateral hydrophobic surface, while the anion of cholic acid has only the convex hydrophobic surface of the steroid skeleton. In the case of cholic acid, the mutual proximity and the same spatial orientation of the C3 and C7 OH groups enable the cooperative binding of water molecules (H-bonds) from the hydration layer in the vicinity of the A ring of the steroid skeleton (Figure 10). Therefore, when building an inclusion complex between βCD and the NaDC anion, there is a greater hydrophobic effect than when building an inclusion complex with the anion of cholic acid. The equilibrium constant for the formation of the inclusion complex βCD-reduced Triton X100 has a value (1:1 stoichiometry) of 145 dm^3^mol^−1^ [57], which is significantly lower than the value in Table 3. Reduced Triton X100 has a movable cyclohexane ring of a chair conformation with a conformationally flexible short alkyl chain, while bile acid anions have the A ring of the steroid skeleton also in a chair conformation; however, *cis* binding of the A ring to the B ring of the steroid skeleton prevents conformational mobility. The conformational mobility probably enables a less efficient dehydration of the hydrophobic molecular surface, and therefore the hydrophobic effect during the formation of the inclusion complex is reduced. The βCD–Triton X100 inclusion complex has a value (1:1 stoichiometry) of 3327 dm^3^mol^−1^ [57], where Triton X100 has a conformationally rigid aromatic ring. The value of 3327 dm^3^mol^−1^ is of the order of magnitude as the equilibrium constant of the formation of the inclusion complex βCD–NaDC (Table 3).

The investigated alkaline metal ions (Na^+^ and Cs^+^) have a synergistic effect on the self-association of cholic acid and deoxycholic acid anions (they reduce CMC); however, on the contrary, they have an antagonistic effect on the value of the equilibrium constant of the formation of inclusion complexes with the investigated bile acid anions (Table 3). Although in both processes (formation of micelles and formation of the inclusion complex); the hydrophobic effect provides a driving force, nevertheless Na^+^ and Cs^+^ ions act differently.

**Figure 11:**
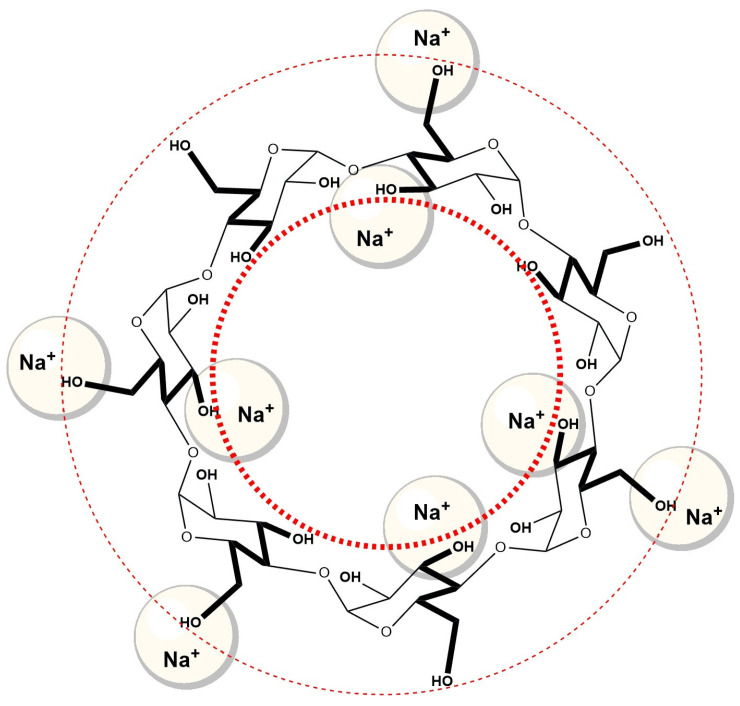
Na^+^ ions with OH groups of β-cyclodextrin (βCD) near the entrance of the hydrophobic cavity build an ion–dipole interaction, which makes it difficult for the A ring of the steroid skeleton sodium deoxycholate (NaDC) and sodium cholate (NaC) to enter.

Na^+^ ions have relatively small volumes compared to Cs^+^ ions, and therefore they have a higher surface density of positive charge and enter into an ion–dipole electrostatic interaction with oxygens (negative dipole of OH groups) of OH groups [58]. Therefore, probably similar to βCD, Na^+^ ions form an ion–dipole interaction with OH groups located in the vicinity of the entrance of the hydrophobic cavity of cyclodextrin (Figure 11). As a result, Na^+^ hinders the entry of the A ring of the steroid skeleton of NaDC and NaC into the hydrophobic cavity of β-cyclodextrin compared to the aqueous solution of βCD, which does not contain an additional amount of Na^+^ ions (solution of NaCl in a concentration of 400 mM).

**Figure 12:**
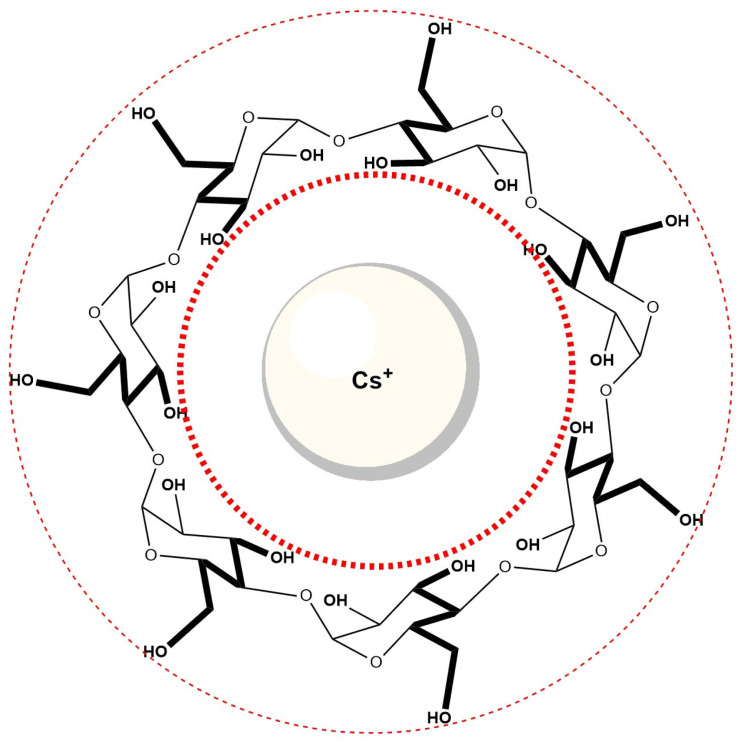
The Cs^+^ ion is probably incorporated into the hydrophobic cavity of cyclodextrin.

Cs^+^ ions, due to their low surface charge density and weak hydration, have been described as “hydrophobic cations,” and may interact with hydrophobic cavities such as that of β-cyclodextrin (Figure 12). It is therefore plausible that Cs^+^ ions partially occupy the βCD cavity, leading to competition with bile salt anions (specifically the A ring of the steroid skeleton) for inclusion. Such competition could explain the observed decrease in the equilibrium constant for complex formation in βCD solutions containing 400 mM CsCl, compared to βCD solutions without added Cs^+^. Although direct evidence for Cs^+^ inclusion is not available in this system, similar effects have been proposed in related host–guest studies involving large alkali metal cations [59].

Both micellization and inclusion complexation processes investigated in this study are predominantly entropy-driven at room temperature; however, the mechanisms by which Na^+^ and Cs^+^ ions influence these processes differ. While sodium ions are moderately hydrated and interact primarily through indirect modulation of water structure, cesium ions, being more chaotropic, may more directly affect hydrophobic aggregation and inclusion equilibria. The findings of this study extend current understanding of the physicochemical behavior of bile salts in complex media. By quantitatively describing the effects of βCD and alkali metal ions on both micellization and inclusion complex formation, the study offers a detailed model of how supramolecular interactions and electrostatic effects can compete or cooperate in self-assembling systems. These insights are particularly relevant for the rational design of bile salt-based drug carriers, where controlling aggregation and host–guest equilibrium is crucial. Moreover, understanding how specific ions influence these interactions contributes to broader knowledge of Hofmeister effects in supramolecular chemistry.

## 4. Materials and Methods

### 4.1. Materials

Substances used in this research were purchased: β-cyclodextrin, 98% (Thermo Scientific, Waltham, MA, USA); sodium deoxycholate, 99% (Alfa Aesar, Haverhill, MA, USA), sodium cholate, 99% (Alfa Aesar, Haverhill, MA, USA), sodium chloride, 99% (Alfa Aesar, Haverhill, MA, USA), cesium chloride, 99% (Alfa Aesar, Haverhill, MA, USA), pyren, 98% (Sigma-Aldrich, St. Louis, MO, USA). All substances were used as received from the manufacturer.

### 4.2. Preparation of Stock and Working Solutions

For tensiometric and conductometric measurements, a stock solution of β-cyclodextrin was prepared by dissolving approximately 4.2562 g of βCD in 250 mL of deionized water at 40–45 °C under magnetic stirring to ensure complete dissolution. Stock solutions of the surfactants—sodium cholate (NaC) and sodium deoxycholate (NaDC)—were prepared at concentrations approximately twice their critical micelle concentrations by dissolving the appropriate amounts of NaC and NaDC in deionized water. Working solutions without βCD were prepared by serial dilution of the surfactant stock solutions with deionized water. Working solutions containing βCD were prepared in the same way, but with the addition of a fixed volume of βCD stock solution, depending on the desired final βCD concentration in each series (2 mM or 3 mM).

All solutions were prepared using deionized water, and the final pH was in the range of 7.0–7.4. This near-neutral pH ensures full ionization of the bile salts and preserves the native state of β-cyclodextrin, thereby minimizing pH-dependent variability in micellization and complex formation. These conditions are also relevant for physiological and pharmaceutical applications.

For fluorometric measurements, pyrene was used as a probe molecule. A stock solution of pyrene in ethanol (1 mg/mL) was prepared, and 120 µL of this solution was transferred into a 1 L volumetric flask. The ethanol was evaporated and deionized water was added to the mark. The solution was stirred for 24 h to ensure equilibration, resulting in a final pyrene concentration of 0.12 mg/L. This pyrene water was used to prepare all stock and working solutions for fluorescence spectroscopy, ensuring a consistent probe concentration across all samples [60].

For the preparation of the NaCl and CsCl series of solutions, the pyrene water was prepared in the same way as described above, with the only difference being the addition of the appropriate amount of salt to achieve a final concentration of 400 mM. This approach ensured that both the pyrene and salt concentrations remained constant across all stock and working solutions within each respective series.

A stock solution of βCD was then prepared (4.2562 g of βCD in 250 mL) using pyrene water (with or without salt) at 40–45 °C, under stirring to ensure full dissolution. Similarly, stock solutions of NaC and NaDC were prepared in pyrene water (with or without salt) at concentrations approximately twice their CMCs. Working solutions without βCD were prepared by dilution of the surfactant stock solutions with pyrene water (with or without salt). Working solutions containing βCD were prepared by adding a fixed volume of the βCD stock solution to each sample, depending on the target βCD concentration (1, 2, 3, 4, or 5 mM).

### 4.3. Tensiometric Measurements

Surface tension measurements were performed at a temperature of 298.15 ± 0.1 K using the du Nouy ring method on a Krüss tensiometer (Hamburg, Germany). Critical micelle concentrations (CMCs) of sodium deoxycholate (NaDC) and sodium cholate (NaC) were determined from the surface tension isotherms as the concentration corresponding to the breakpoint, beyond which no further decrease in surface tension (γ) was observed with additional surfactant. Trials were repeated five times for reproducibility. The relative error of the CMC determination never exceeded 5%.

### 4.4. Conductometric Measurements

The data were acquired using a Consort C 860 conductometer (Turnhout, Belgium). The cell containing solutions was immersed in a water bath, controlling the temperature variation at ±0.1 °C. The temperature was kept constant at 298.15 K.

### 4.5. Spectrofluorometric Measurements

Spectrofluorometric measurements were performed on a Carry Eclipse device (Agilent, Waldbronn, Germany). Pyrene was excited at a wavelength of 334 nm. The intensity ratio of the first (373 nm) and third (383 nm) vibration bands was monitored as a function of the surfactant concentration at a temperature of 298.15 K. From the experimental data, values of the critical micellar concentrations of surfactants were determined by Boltzmann fitting in the OriginLab 9 program. Trials were repeated five times for reproducibility. The relative error of the CMC determination never exceeded 5%.

## 5. Conclusions

This study demonstrates that β-cyclodextrin significantly influences the micellization behavior of sodium deoxycholate (NaDC) and sodium cholate (NaC) through the formation of inclusion complexes in aqueous solutions. The observed increase in critical micelle concentration (CMC) with rising βCD concentrations confirms a competitive relationship between micellization and complexation processes. The effect is more pronounced for NaDC due to its greater hydrophobic surface area, which promotes stronger interactions with the βCD cavity. Additionally, the presence of salts such as NaCl and CsCl decreases the CMC, a synergistic effect on the process of self-association. However, both salts also influence the complexation process by altering the effective availability of βCD molecules in solution, an antagonistic effect on the process of formation of the inclusion complex. The equilibrium constants determined for inclusion complex formation provide quantitative support for the differential binding affinities of βCD toward dihydroxy and trihydroxy bile salts. Overall, this work contributes to a deeper understanding of the interplay between surfactant aggregation, host–guest interactions, and ionic environment, insights that are essential for the rational design of drug delivery systems and other supramolecular applications involving bile salts and cyclodextrins.

## Figures and Tables

**Table 1:** Experimentally obtained CMC values of NaDC and NaC in aqueous solutions with βCD at 298.15 K.

c(βCD) mM	CMC/mM	
	NaDC	NaC
0	5.2841 ± 0.1585	12.4215 ± 0.0186
2	7.2151 ± 0.1804	13.6063 ± 0.2041
3	8.1113 ± 0.1622	15.5770 ± 0.2336

Errors represent standard deviations from five independent measurements.

**Table 2:** CMC values of NaDC and NaC in aqueous solution with βCD, at 298.15 K.

βCD c/mM	CMC/mM					
	NaDC in Aqueous Solution	NaDC in 400 mM NaCl Solution	NaDC in 400 mM CsCl Solution	NaC in Aqueous Solution	NaC in 400 mM NaCl Solution	NaC in 400 mM CsCl Solution
0	5.4369 ± 0.2447	1.8172 ± 0.0818	1.5711 ± 0.0707	13.0390 ± 0.3260	6.1940 ± 0.1858	4.9165 ± 0.1967
1	6.3765 ± 0.2869	2.1181 ± 0.0953	2.8144 ± 0.1126	13.3098 ± 0.3256	6.8449 ± 0.2053	6.0794 ± 0.2128
2	7.4155 ± 0.29662	2.6969 ± 0.0944	3.6933 ± 0.1108	13.7389 ± 0.3435	7.3947 ± 0.2218	6.9549 ± 0.2434
3	8.2798 ± 0.2898	4.6993 ± 0.1410	4.0013 ± 0.1200	15.7751 ± 0.3255	7.9859 ± 0.1996	7.6025 ± 0.2281
4	9.3441 ± 0.2803	4.8034 ± 0.1441	4.5442 ± 0.1363	17.3762 ± 0.3475	9.2951 ± 0.2324	8.1205 ± 0.2436
5	10.1349 ± 0.3040	6.1963 ± 0.1549	5.5427 ± 0.2771	17.3564 ± 0.3471	11.0907 ± 0.2218	8.6053 ± 0.2582

Errors represent standard deviations from five independent measurements.

**Table 3:** Equilibrium constants $K_{\left[\beta CD-S\right]}$ of sodium deoxycholate (NaDC) and sodium cholate (NaC) in aqueous solution, 400 mM NaCl solution, and 400 mM CsCl solution at 298.15 K.

$K_{\left[\beta CD-S\right]}$ (dm^3^/mol)					
NaDC in Aqueous Solution	NaDC in 400 mM NaCl Solution	NaDC in 400 mM CsCl Solution	NaC in Aqueous Solution	NaC in 400 mM NaCl Solution	NaC in 400 mM CsCl Solution
3703.70 ± 240.74	2610.97 ± 156.66	3039.51 ± 197.57	1040.58 ± 67.64	904.16 ± 58.77	923.36 ± 60.02

Errors represent standard deviations from five independent measurements.

## Data Availability

The original data presented in the study are openly available in Supplementary Materials.

## Supplementary Materials

The following supporting information can be downloaded at https://www.mdpi.com/article/10.3390/molecules30102197/s1. Table S1: Data obtained by spectrofluorimetric measurements of NaC in aqueous solutions with different βCD concentrations; Table S2: Data obtained by spectrofluorimetric measurements of NaC in 400 mM NaCl solutions with different βCD concentrations; Table S3: Data obtained by spectrofluorimetric measurements of NaC in 400 mM CsCl solutions with different βCD concentrations; Table S4: Data obtained by spectrofluorimetric measurements of NaDC in aqueous solutions with different βCD concentrations; Table S5: Data obtained by spectrofluorimetric measurements of NaDC in 400 mM NaCl solutions with different βCD concentrations; Table S6: Data obtained by spectrofluorimetric measurements of NaDC in 400 mM CsCl solutions with different βCD concentrations; Figure S1: Change in specific conductivity as a function of sodium deoxycholate in aqueous solution; Figure S2: Change in specific conductivity as a function of sodium deoxycholate in 2 mM βCD solution; Figure S3: Change in specific conductivity as a function of sodium cholate in aqueous solution.

## Abbreviations

The following abbreviations are used in this manuscript:

βCD	beta cyclodextrin
NaDC	sodium deoxycholate
NaC	sodium cholate
CMC	critical micellar concentration
