# Supplementary material for: Effect of β-Cyclodextrin on the Aggregation Behavior of Sodium Deoxycholate and Sodium Cholate in Aqueous Solution
Source: Molecules. 2025 May 17;30(10):2197. doi: 10.3390/molecules30102197 (PMC12113708; doi:10.3390/molecules30102197)
Supplement: Supplementary file 1 [file molecules-30-02197-s001.zip › molecules-3630101-supplementary.pdf]

Supplementary Material

Table S1. Data obtained by spectrofluorimetric measurements of NaC in aqueous solutions with different βCD concentrations

0 mM βCD		1 mM βCD		2 mM βCD		3 mM βCD		4 mM βCD		5 mM βCD	
c(NaC) mM	I_1/I_3	c(NaC) mM	I_1/I_3	c(NaC) mM	I_1/I_3	c(NaC) mM	I_1/I_3	c(NaC) mM	I_1/I_3	c(NaC) mM	I_1/I_3
1	1.77099	8	1.697	6	1.7211	12	1.5836	6	1.5846	8	1.5923
4	1.7712	9	1.6968	8	1.6986	13	1.5112	8	1.5746	10	1.5729
8	1.6752	10	1.6600	10	1.6964	14	1.4172	10	1.5418	12	1.5881
12	1.4353	11	1.6204	12	1.5112	15	1.4012	12	1.5032	13	1.6178
16	1.105	12	1.5007	14	1.2684	16	1.2939	14	1.4719	14	1.4868
20	0.9912	13	1.4005	16	1.1321	17	1.1880	16	1.3665	15	1.6232
24	0.9433	14	1.2691	18	1.0397	18	1.0766	18	1.1245	16	1.4239
28	0.8776	15	1.1956	20	0.9846	19	1.0413	20	1.0560	17	1.3233
9	1.6671	16	1.1406	25	0.9190	21	1.00388	25	0.9362	18	1.1913
10	1.6079	17	1.1377	30	0.8759	23	0.93198	30	0.8838	19	1.0969
13	1.3455	18	1.0396			25	0.9142	35	0.8523	21	1.0257
14	1.2664	19	1.0097			27	0.8907	40	0.8466	23	0.9469

Table S2. Data obtained by spectrofluorimetric measurements of NaC in 400 mM NaCl solutions with different βCD concentrations

0 mM βCD		1 mM βCD		2 mM βCD		3 mM βCD		4 mM βCD		5 mM βCD	
c(NaC) mM	I_1/I_3	c(NaC) mM	I_1/I_3	c(NaC) mM	I_1/I_3	c(NaC) mM	I_1/I_3	c(NaC) mM	I_1/I_3	c(NaC) mM	I_1/I_3
8	0.8446	1	1.7337	9	0.9000	3	1.7107	3	1.6358	4	1.6116
9	0.8295	3	1.7245	10	0.8240	4	1.6780	4	1.6550	8	1.6323
10	0.8372	5	1.7321	11	0.8503	5	1.6664	5	1.7178	12	0.9441
11	0.8079	7	1.2566	12	0.8156	6	1.6742	6	1.6771	16	0.8309
0.25	1.7797	8	1.2251	5	1.7647	7	1.6176	0.5	1.3812	9	1.5477
1	1.7833	9	1.1473	6	1.6144	8	1.3220	0.75	1.4249	10	1.1623
3	1.7711	10	0.9750	7	1.4455	9	1.0679	1	1.4690	10.5	1.2209
5	1.5334	11	0.948	8	1.1029	10	0.9392	2	1.5859	11	1.1056
5.5	1.5729	5.5	1.6954	8.2	1.1025	11	0.8592	7	1.7011	9.25	1.7357
6	1.3555	6	1.5789	8.4	0.9577	12	0.8475	8	1.6031	9.5	1.7255
6.5	1.3480	6.5	1.4204	8.6	0.9601	7.5	1.2856	9	1.3301	9.75	1.6982
7	1.4251	0.5	1.8428	8.8	0.9871	8.5	1.1159	10	1.1258	12	1.5606
								11	1.0142		
								12	0.9325		
								13	0.9028		
								14	0.8712		

Table S3. Data obtained by spectrofluorimetric measurements of NaC in 400 mM CsCl solutions with different βCD concentrations

0 mM βCD		1 mM βCD		2 mM βCD		3 mM βCD		4 mM βCD		5 mM βCD	
c(NaC) mM	I_1/I_3	c(NaC) mM	I_1/I_3	c(NaC) mM	I_1/I_3	c(NaC) mM	I_1/I_3	c(NaC) mM	I_1/I_3	c(NaC) mM	I_1/I_3
0.25	1.7751	1	1.7423	3	1.6725	3	1.6041	3	1.4065	3	1.4776
1	1.7734	3	1.7683	5	1.6314	5	1.6473	5	1.4643	5	1.5477
3	1.7788	5	1.6896	6	1.4453	6	1.4977	6	1.5322	6	1.5499
5	1.3498	7	1.0864	7	1.1591	7	1.3035	7	1.5242	7	1.5283
3.5	1.6821	5.5	1.5393	5.5	1.7143	5.5	1.6165	8	1.1845	8	1.2744
4	1.5726	6	1.4130	6.5	1.4497	6.5	1.4759	9	0.9653	9	1.0548

5.25	1.1519	6.5	1.3449	7.5	1.0670	7.5	1.2398	10	0.9237	10	0.9560
5.5	1.1048	8	1.1303	8	1.0213	8	1.2067	11	0.8025	11	0.8365
6	1.0047	2	1.7731	1	1.3684	1	1.5687	7.5	1.2504	7.5	1.281
7	0.8983	4	1.7214	2	1.608	2	1.6690	2	1.171	2	1.5006
8	0.8605	5.75	1.5285	9	0.8803	9	0.9051	12	0.8403	12	0.8283
9	0.8379	6.75	1.3191	10	0.8493	10	0.8620	13	0.7931	13	0.8334

Table S4. Data obtained by spectrofluorimetric measurements of NaDC in aqueous solutions with different βCD concentrations

0 mM βCD		1 mM βCD		2 mM βCD		3 mM βCD		4 mM βCD		5 mM βCD	
c(NaDC) mM	I_1/I_3	c(NaDC) mM	I_1/I_3	c(NaDC) mM	I_1/I_3	c(NaDC) mM	I_1/I_3	c(NaDC) mM	I_1/I_3	c(NaDC) mM	I_1/I_3
0.5	1.7831	0.5	1.7194	0.5	1.6516	0.5	1.4624	4	1.6043	4	1.6064
1	1.7441	1	1.7464	1	1.6986	1	1.5024	5	1.6068	5	1.6632
2	1.7199	2	1.7687	2	1.7438	2	1.6273	6	1.6399	6	1.7175
3	1.6716	3	1.7140	3	1.7544	3	1.6182	7	1.6388	7	1.6847
4	1.5934	4	1.7086	4	1.7627	4	1.7753	8	1.6459	8	1.5916
5	1.4451	5	1.5914	5	1.7366	5	1.7513	9	1.2566	9	1.5601
6	0.9988	6	1.4321	6	1.6746	6	1.6888	10	0.9871	10	1.2501
7	0.8928	7	1.0295	7	1.4486	7	1.6342	11	0.8178	11	1.3258
8	0.7983	8	0.8275	8	0.9807	8	1.3603	12	0.7571	12	1.4939
9	0.7480	9	0.9403	9	0.8487	9	0.9921	13	0.7390	13	1.5783
10	0.7390	10	0.7639	10	0.7557	10	0.8366	14	0.7165	14	0.7403
11	0.7374	11	0.7269	11	0.7714	11	0.7559	15	0.7251	15	0.7408

Table S5. Data obtained by spectrofluorimetric measurements of NaDC in 400 mM NaCl solutions with different βCD concentrations

0 mM βCD		1 mM βCD		2 mM βCD		3 mM βCD		4 mM βCD		5 mM βCD	
c(NaDC) mM	I_1/I_3	c(NaDC) mM	I_1/I_3	c(NaDC) mM	I_1/I_3	c(NaDC) mM	I_1/I_3	c(NaDC) mM	I_1/I_3	c(NaDC) mM	I_1/I_3
0.25	1.7868	1	1.7476	3	0.8734	4	1.1307	4	1.7120	6.7	0.7891
0.5	1.7984	2	1.7150	4	0.7472	5	0.7278	5	1.0043	6	0.9255
0.75	1.7617	3	0.7527	5	0.7186	6	0.8111	6	0.7339	5.6	1.2883
1	1.70177	4	0.7498	6	0.7137	7	0.7286	7	0.7382	5.2	1.4741
1.5	1.1837	2.2	0.8220	1	1.6901	3	1.6649	8	0.7214	4.8	1.5838
2	0.8418	2.4	0.8093	1.5	1.7308	2	1.4823	9.31	0.6834	4.4	1.5695
2.5	0.7217	2.6	0.7955	2	1.7223	2.5	1.5382	2	1.6517	4	1.5226
3	0.7245	2.8	0.7241	2.5	1.3869	3.5	1.5794	3	1.7031	3.6	1.4845
1.25	1.7457	0.5	1.7289	2.6	1.1722	3.6	1.5043	4.2	1.5848	5.8	1.1864
1.75	1.6968	0.75	1.7324	2.8	1.3068	3.8	1.6672	4.4	1.3715	5.4	1.4600
4	0.7114	5	0.7376	1	1.6390	4.25	1.6633	4.6	1.6895	6.2	1.4686
5	0.6893	6	0.7244	2	1.7112	4.5	1.6756	4.8	1.0390	6.4	1.1944
										7	0.7105
										8	0.7343
										9	0.7211
										10	0.6911

Table S6. Data obtained by spectrofluorimetric measurements of NaDC in 400 mM CsCl solutions with different βCD concentrations

0 mM βCD		1 mM βCD		2 mM βCD		3 mM βCD		4 mM βCD		5 mM βCD	
c(NaDC) mM	I_1/I_3	c(NaDC) mM	I_1/I_3	c(NaDC) mM	I_1/I_3	c(NaDC) mM	I_1/I_3	c(NaDC) mM	I_1/I_3	c(NaDC) mM	I_1/I_3
0.25	1.7646	0.25	1.6805	0.5	1.4870	0.5	1.3049	4	1.5743	3	1.3411
0.5	1.8298	0.5	1.6837	1	1.5705	1	1.4258	5	0.7534	4	1.5712
0.75	1.6898	0.75	1.7474	2	1.7086	2	1.6002	6	0.7037	4.5	1.5389

1	1.7243	1	1.7462	3	1.5740	3	1.6387	7	0.7051	5	1.3335
2	0.8525	2	1.5804	4	0.7213	4	1.1374	4.1	1.4095	6	0.8258
2.5	0.7398	3	1.0021	5	0.7177	5	0.7459	4.3	1.3192	7	0.7268
3	0.7263	8	0.7021	6	0.7368	6	0.723	4.5	1.1149	8	0.7228
1.75	0.9375	10	0.7116	7	0.6892	7	0.6969	4.7	0.9936	9	0.7048
2.25	0.7581	2.2	1.5194	3.2	1.7091	2.5	1.5904	3.25	1.4792	5.2	1.3790
4	0.7225	2.4	1.2971	3.4	1.5542	3.5	1.4454	3.5	1.4514	5.4	1.1410
5	0.7040	2.6	1.4324	3.6	1.3986	4.5	0.7954	3.75	1.5275	5.6	0.8336
1.5	1.3091	2.8	1.3286	3.8	1.025	4.2	0.8767	3.9	1.4683	5.8	1.2398

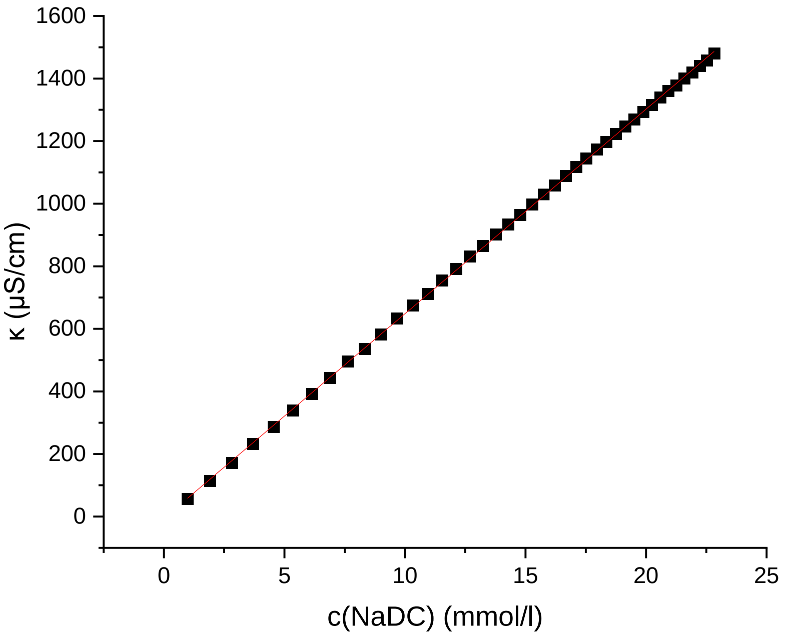

Figure S1. Change of specific conductivity as a function of sodium-deoxycholate in aqueous solution

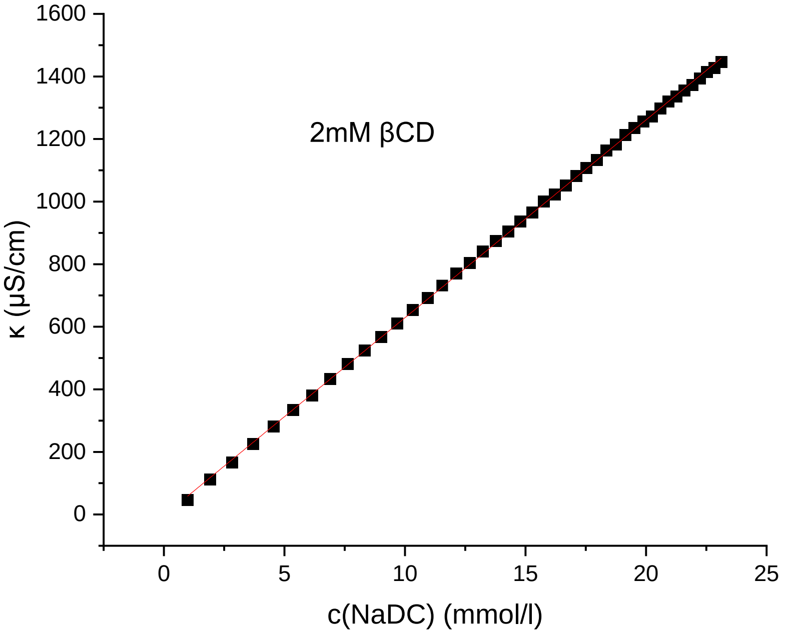

Figure S2. Change of specific conductivity as a function of sodium-deoxycholate in 2 mM βCD solution

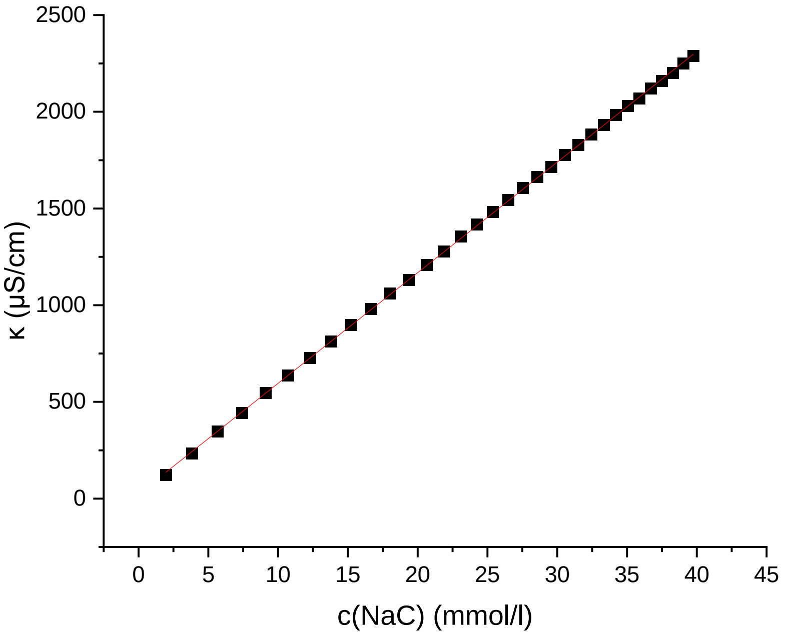

Figure S3. Change of specific conductivity as a function of sodium-cholate in aqueous solution
